# Placebo Effect in the Treatment of Depression and Anxiety

**DOI:** 10.3389/fpsyt.2019.00407

**Published:** 2019-06-13

**Authors:** Irving Kirsch

**Affiliations:** Harvard Medical School, Boston, MA, United States

**Keywords:** placebo, nocebo, depression, anxiety, antidepressants

## Abstract

The aim of this review is to evaluate the placebo effect in the treatment of anxiety and depression. Antidepressants are supposed to work by fixing a chemical imbalance, specifically, a lack of serotonin or norepinephrine in the brain. However, analyses of the published and the unpublished clinical trial data are consistent in showing that most (if not all) of the benefits of antidepressants in the treatment of depression and anxiety are due to the placebo response, and the difference in improvement between drug and placebo is not clinically meaningful and may be due to breaking blind by both patients and clinicians. Although this conclusion has been the subject of intense controversy, the current article indicates that the data from all of the published meta-analyses report the same results. This is also true of recent meta-analysis of all of the antidepressant data submitted to the Food and Drug Administration (FDA) in the process of seeking drug approval. Also, contrary to previously published results, the new FDA analysis reveals that the placebo response has not increased over time. Other treatments (e.g., psychotherapy and physical exercise) produce the same benefits as antidepressants and do so without the side effects and health risks of the active drugs. Psychotherapy and placebo treatments also show a lower relapse rate than that reported for antidepressant medication.

## Introduction

The aim of this review is to evaluate the placebo effect in the treatment of anxiety and depression. On February 19, 2012, Leslie Stahl opened a segment of the CBS news program *60 Minutes* saying “The medical community is at war, battling over the scientific research and writings of a psychologist named Irving Kirsch. The fight is about antidepressants and Kirsch’s questioning of whether they work.” By that time, I had co-authored three meta-analyses and a book concerning the placebo effect in the treatment of depression (1–4). Two of these meta-analyses (2, 3) were conducted on the data sent to the Food and Drug Administration (FDA) by the manufacturers of what at that time were the six most widely prescribed antidepressants—data that we obtained using the Freedom of Information Act. We found that although the people given antidepressants showed considerable improvement in the clinical trials submitted to the FDA by the manufacturers, so did the people given placebo, and the difference in outcome between drug and placebo was below the criterion for clinical meaningfulness used by the National Institute for Health and Care Excellence (NICE), the organization that sets treatment guidelines for the National Health Service in the United Kingdom.

There is now a crisis concerning the lack of replicability of many studies in psychology and medicine (5, 6). I am pleased to report that the antidepressant meta-analyses we published have not contributed to this crisis. There are now at least nine subsequent meta-analyses aimed at replicating or discrediting our studies (7–16). Some of these were restricted to changes on the Hamilton Rating Scale for Depression (HAM-D), whereas others included data from a variety of scales. Some were conventional meta-analyses in which means and standard deviations were used to calculate effect sizes, whereas others were patient-level analyses. Although interpretations of the data varied from study to study, the results have been consistent across all of them. We had reported a mean drug-placebo difference of 1.80 points on the HAM-D and a standardized mean difference (SMD) of 0.32. The differences reported in the replications ranged from 1.62 to 2.56 HAM-D points, with SMD effect sizes ranging from 0.23 to 0.34. To put this into perspective, the NICE criteria for clinical significance of antidepressant-placebo differences are three points on the HAM-D or SMDs of at least 0.50, corresponding to what Cohen (17) proposed as a moderate effect size.

Special attention is due to the preliminary results of a patient-level meta-analysis reported by Stone et al. (15). Marc Stone is the Deputy Director for Safety at the Division of Psychiatric Products of the FDA. He and his colleagues reported a patient-level analysis of the data from all randomized placebo-controlled trials of antidepressants in the treatment of Major Depressive Disorder that had been submitted to the FDA between 1979 and 2016. The similarity in outcome between what the Stone et al. data and those that my colleagues and I had reported in 2002 and 2008 is astounding. We had reported a drug response of 10.1 points on the HAM-D and a placebo response of 8.3 point—a drug-placebo difference of 1.8 points. In Stone et al.’s comprehensive analysis of the data from the 73,178 patients in the 228 trials submitted to the FDA, the drug response was 10.1 points, the placebo response was 8.3 points—yielding a drug-placebo difference of 1.80 points on the 17-item HAM-D, exactly what my colleagues and I reported in our analysis of the FDA data for the six antidepressants that we evaluated (2).

Antidepressants are also used to treat anxiety disorders. Might they be more effective in treating anxiety than in treating depression? My colleagues and I have assessed that issue in a meta-analysis of the effects of paroxetine in treating anxiety disorders (18). We chose to limit our analysis to paroxetine so that we could assess a complete dataset of unpublished pre- and post-marketing trials, as well as those that had been published. As part of a 2004 lawsuit settlement, GlaxoSmithKline was required to post online the results of all clinical trials involving its drugs on its Clinical Trial Register (19). Examining these data, we found a drug-placebo effect size (SMD) of 0.27, similar to those reported for antidepressants in the treatment of depression. In a subsequent study, Roest et al. (20) analyzed data obtained from the FDA for premarketing trials of nine second-generation antidepressants in the treatment of anxiety disorders. They reported an SMD of 0.33, similar to that reported by Sugarman and colleagues for paroxetine (18) and to those reported in the meta-analyses of antidepressants in the treatment of depression cited above. Subsequently, Sugarman and colleagues (21) replicated the Roest et al. study and found an SMD of 0.34 across all antidepressants and all anxiety disorders, with individual effect sizes ranging from 0.26 to 0.39. Thus, antidepressants are no better in treating anxiety disorders than they are in treating depression.

The impact of placebo factors in the treatment of anxiety can also be seen in a study by Faria et al. (22). Participants diagnosed with social anxiety disorder (SAD) were treated with an selective seratonin reuptake inhibitor (SSRI) (escitalopram). Approximately half of the patients were accurately informed that they were taking an SSRI. The others were told that they were being given an active placebo (i.e., a drug that produces side effects but has no therapeutic effect on the condition being treated). Telling patients that they were being treated by an active medication doubled its effectiveness on a continuous measure of anxiety and tripled the response rate.

Critics have noted that the criteria proposed for clinical significance by NICE (3 points on the HAM-D or SMDs of at least 0.50) are arbitrary (23), and they are correct. The NICE criteria are as arbitrary as the criterion of p < .05 for statistical significance, the use of a 50% reduction in symptoms as a criterion of a clinical response, and the use of a HAM-D score below 8 as the criterion of remission. Given that the conventional cutoffs for statistical significance are arbitrary, as are those for assessing clinical “response” and “remission,” why would we expect the criteria for the clinical significance of drug-placebo differences to be any less arbitrary?

Nevertheless, Joanna Moncrieff and I (24) have proposed empirically derived criteria for the clinical significance of antidepressant-placebo differences. We used published data from a large patient-level analysis (25) of the correspondence between changes on the HAM-D and the Clinical Global Impressions-Improvement (CGI-I) scale, a scale that rates improvement on a scale of 1 (very much improved) through 4 (no change) to 7 (very much worse). This analysis revealed that an improvement of three points on the HAM-D (SMD = 0.375) is equivalent to a clinician rating “no change” on the CGI-I. A CGI-I rating of “minimally improved” corresponds to a HAM-D difference of 7 points (SMD = 0.873), and a rating of “much improved” corresponds to a 14-point HAM-D difference (SMD = 1.75). None of the meta-analyses have reported drug-placebo differences that come close to reaching the criterion for CGI-I ratings of minimal improvement, even among the most severely depressed patients.

Many depressed patients report substantial improvement after taking antidepressant medication, as do psychiatrists when describing their outcomes. How are we to reconcile this with the consistent finding that the differences between the response to antidepressants and placebos are vanishingly small? The answer is the placebo response. Although drug–placebo differences in outcome are equivalent to no difference at all, both drug and placebo responses can be substantial. The improvement of 8.3 points following placebo treatment and 10.1 points on the active drugs reported by Kirsch et al. (3) and Stone et al. (15) corresponds to CGI-I ratings between minimally improved and much improved. It is only the 1.8-point difference that corresponds to a CGI-I rating of no change. Thus, the clinically meaningful improvement seen following prescriptions of antidepressants is largely to the placebo response (i.e., the placebo effect, regression toward the mean, and spontaneous remission).

The failure to find meaningful differences between antidepressants and placebos has been blamed on increasing placebo responses over the years (26), and some meta-analyses have shown increases in both the placebo response and the drug response over time [e.g., Ref. (27)]. However, the comprehensive analysis of all trials submitted to the FDA from 1979 to 2016 tells a different story (15). The placebo response was 8.3 HAM-D points in both 1979 and 2016, with little variation between those dates. There was a small decrease (0.8 points) in the drug–placebo difference over time, but this was due to a 0.8-point decrease in the drug response (from 10.7 points in 1979 to 9.9 points in 2016), rather than an increase in the placebo response.

## Placebo Effects versus Placebo Responses

In 1965, Fisher and colleagues (28, pp. 57–58) noted that “a clinical response following treatment (*drug response*) is not synonymous with an effect which can be attributed to the treatment (*drug effect*).” In 1998, Kirsch and Sapirstein (4) extended this distinction to placebo responses and effects, and in 2018, a group of 29 internationally recognized placebo researchers published a “consensus statement,” in which they endorsed the view that “the placebo and nocebo response includes all health changes that result after administration of an inactive treatment (i.e., differences in symptoms before and after treatment), thus, including natural history and regression to the mean. The *placebo and nocebo effect* refers to the changes specifically attributable to placebo and nocebo mechanisms” (29, p. 206). The meta-analyses described above indicate a strong placebo response, but with one exception: they do not assess the placebo effect.

In the one exception (4), Guy Sapirstein and I assessed the placebo effect by comparing the placebo response in drug trials to changes observed in no-treatment natural-history control conditions in psychotherapy studies. We found that 25% of the drug response was duplicated in the no-treatment groups, and 75% of the drug response was found in the placebo groups. Thus, the placebo effect was 50% of the drug response—double the drug effect and also double the response found in the no-treatment controls. It was a genuine placebo effect.

A limitation of our study was that data in the no-treatment groups and data in the placebo groups came from different studies. That limitation has been overcome in a clinical trial reported by Leuchter and his colleagues (30). This was a three-arm study, in which depressed patients were randomized to either antidepressant plus supportive care, placebo plus supportive care, or supportive care alone. Mean HAM-D improvement was 10.05 points in the antidepressant group and 7.59 in the placebo group, but only 1.37 in the supportive care only group. As in the Kirsch and Sapirstein study, the response in the placebo group was mostly a genuine placebo effect and not simply due to spontaneous improvement or regression toward the mean.

## Is There a Drug Effect at All?

Although the difference between antidepressant and placebo is not clinically meaningful, it is statistically significant. Can we interpret that small but statistically significant difference as a genuine drug? Although that cannot be ruled out, there is another possibility. Clinical trials in which patients and/or their doctors or other outcome raters are asked to judge whether the patient was given an active drug or a placebo are consistent in showing that those judgements are very accurate. This indicates that the trials are not really double-blind. Numerous studies have shown that when patients know they are getting a drug, they are more responsive to the drug than when they know they might be getting a placebo (31–35). This indicates a placebo effect component in the drug response. Similarly, the placebo response is reduced when people know they might be getting a placebo than when they are led to believe that they are getting the active drug (31, 36). Therefore, the small drug–placebo difference in outcome might be due to the increased response in the drug group and decreased responding in the placebo group produced by what participants are told about the trials.

**Figure 1:**
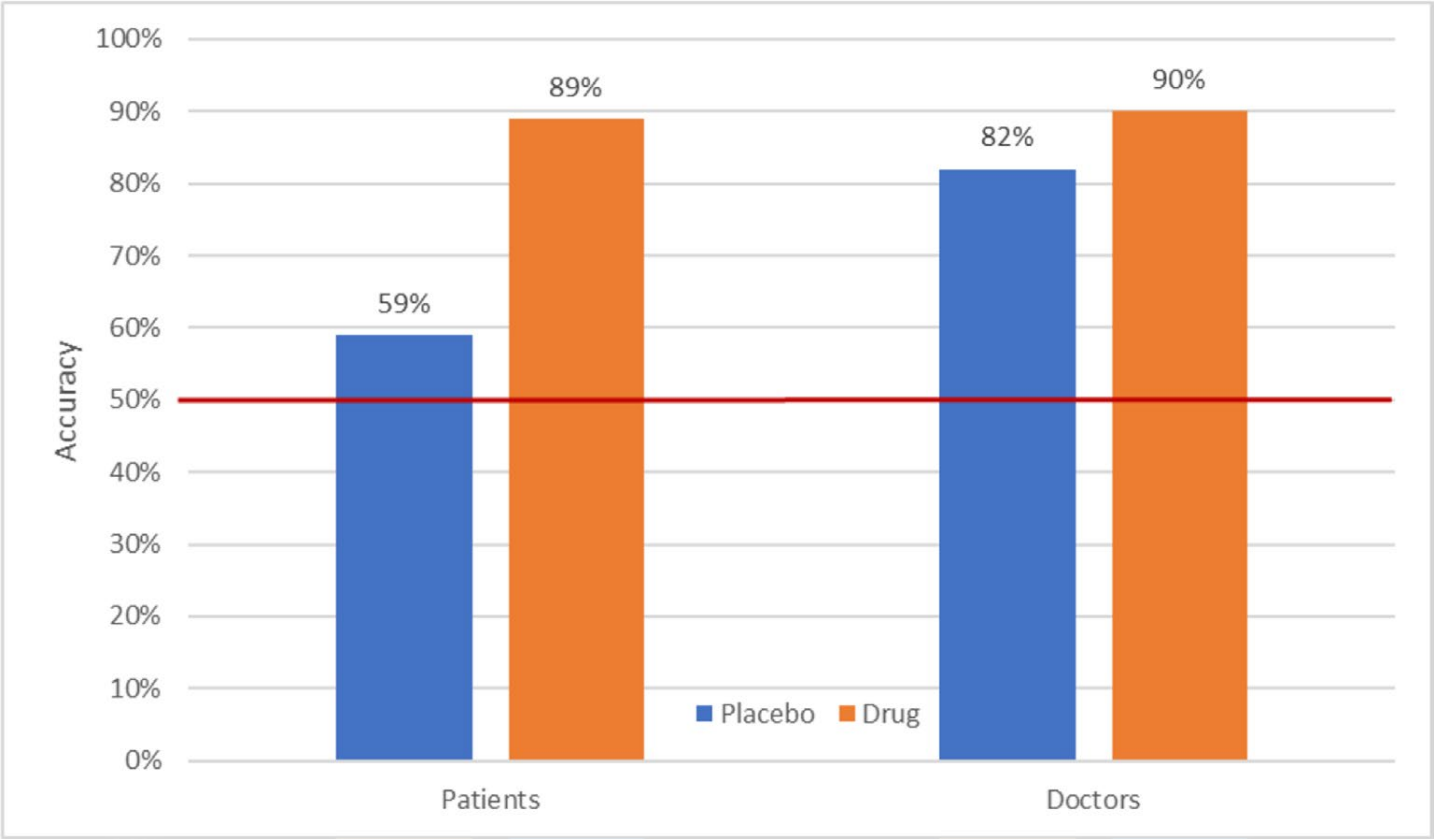
Accuracy of patient and doctor “guesses” as a function of actual treatment (37).

In 1986, Rabkin and her colleagues (1986) published a study in which doctors and their depressed patients who had been randomized to imipramine, phenelzine, or placebo were asked to guess the group to which the patients had been assigned. Overall, 78% of patients and 87% of the doctors accurately identified whether the patients had been given an active drug or a placebo. As shown in **Figure 1**, patients randomized to active drug groups were especially successful in breaking blind, whereas those receiving placebo seem to be merely guessing. In contrast, doctors showed high levels of accuracy in identifying group assignment for patients in the placebo groups as well as those in the drug groups. Furthermore, this pattern of results has been replicated successfully in subsequent studies (38–41), indicating that they are reliable. Rabkin et al. concluded that “in view of these findings we recommend that investigators routinely record and report doctor and patient opinions about treatment assignment in randomized trials, preferably both early in the trial and at the end” (p. 86). Unfortunately, this recommendation has been largely ignored.

Given these exceptionally high rates of breaking blind, the next question is whether this phenomenon is associated with the outcome of clinical trials. In 2013, Baethge and colleagues (42) reported the results of a meta-analysis addressing this issue. In 47 clinical trials of psychiatric disorders in which blinding was assessed, the correlation between patient accuracy and the drug–placebo effect size was .51 (p = .002) and that between rater accuracy and effect size was .55 (p = .067). Thus, the greater the likelihood of breaking blind, the greater the drug–placebo difference.

**Figure 2:**
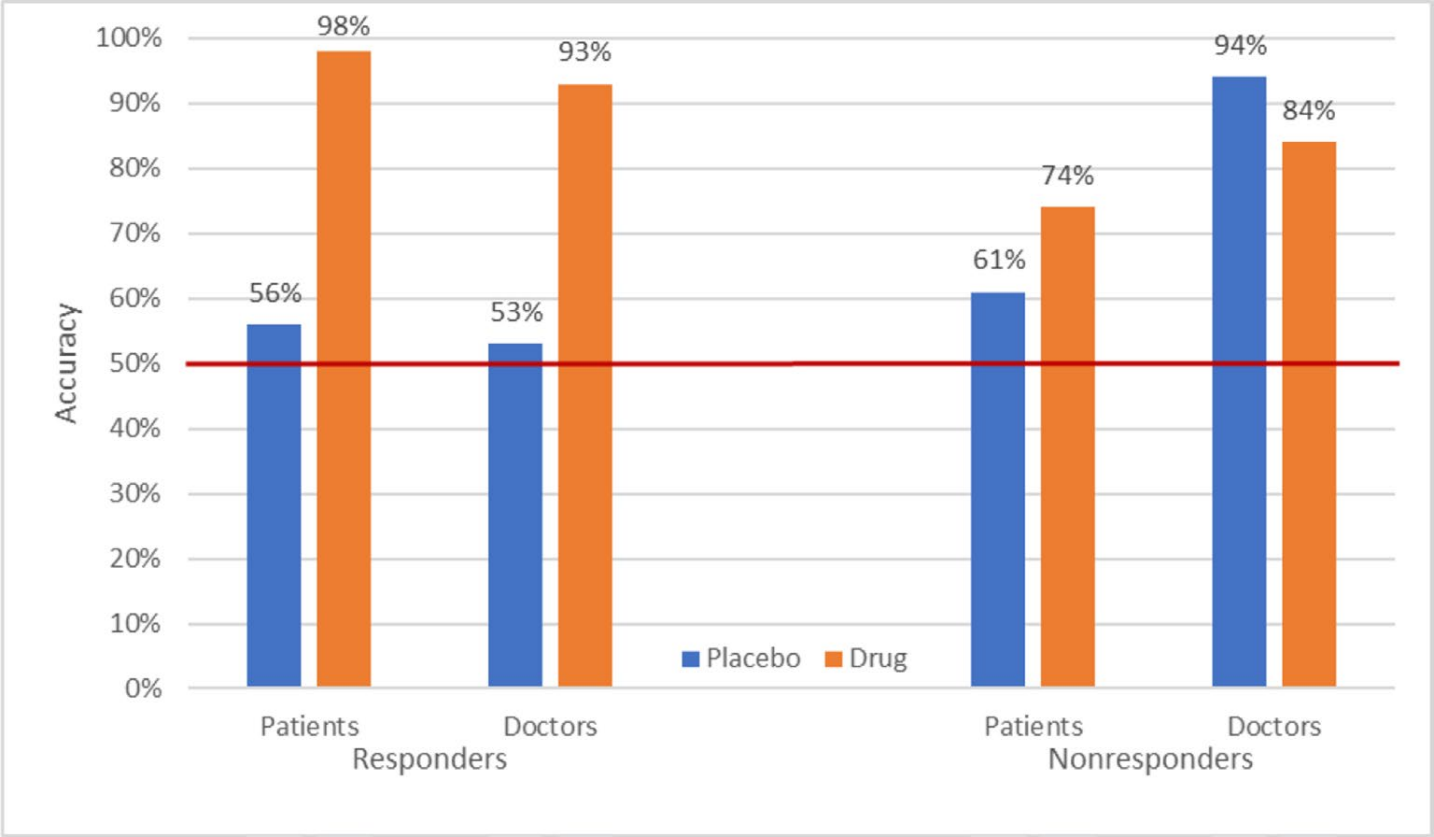
Accuracy of patient and doctor “guesses” as a function of actual treatment and patient response (37).

However, there is an interpretive problem with respect to understanding the direction of causality in the data on accuracy of judgements of group assignment. In most of the studies in which blinding was assessed, the assessment was made near the end of the trial. Thus, it is possible that breaking blind is a consequence rather than a cause of drug–placebo differences. However, some of the data reported by Rabkin et al. (37) indicate that breaking blind is not solely a consequence of the patients’ responses to treatment. **Figure 2** displays the accuracy of judgements separately for patients who responded to treatment and those who did not. Of particular interest is the ability of both patients and doctors to accurately guess group assignment of nonresponders in the drug group. Seventy-four percent of nonresponders who received an active drug judged themselves to be on the drug, as did 84% of their doctors. Furthermore, almost half of responders to placebo guessed they were on placebo. Although this would be expected by chance guessing, it indicates that the improvement experienced by these placebo responders did not lead them to think they were taking an active medication. Taken together, these data indicate that although response to treatment influences patients’ and doctors’ judgements of treatment assignment, it does not fully explain the accuracy of those judgements.

I and others (1, 43, 44) have hypothesized that the presence of side effects is responsible for breaking blind. As part of the informed consent processes, patients in clinical trials are told that they might receive a placebo. They are also told that the medication under investigation has side effects, and they are told exactly what the known side effects are. Now placebos can also generate side effects, a phenomenon known as the nocebo effect, but they do so to a much lesser degree than active medications (45). This difference in side effects might lead patients in clinical trials, as well as the clinicians who rate their improvement, to figure out to which group they have been randomized. To the extent that this occurs, the trial is not really double-blind. In this section, I describe data indicating that patients in clinical trials often do break blind and that breaking blind affects the outcomes of the trials.

Studies have shown mixed results for the hypothesis and drug–placebo differences are associated with reported side effects (46–51). However, side effects may be only one of the cues leading participants in clinical trials to break blind. Joanna Moncrief (52) has hypothesized that people learn how to recognize the sometimes subtle changes produced by medications without necessarily reporting symptoms that would be listed as a side effect on the checklists used to assess them.

**Figure 3:**
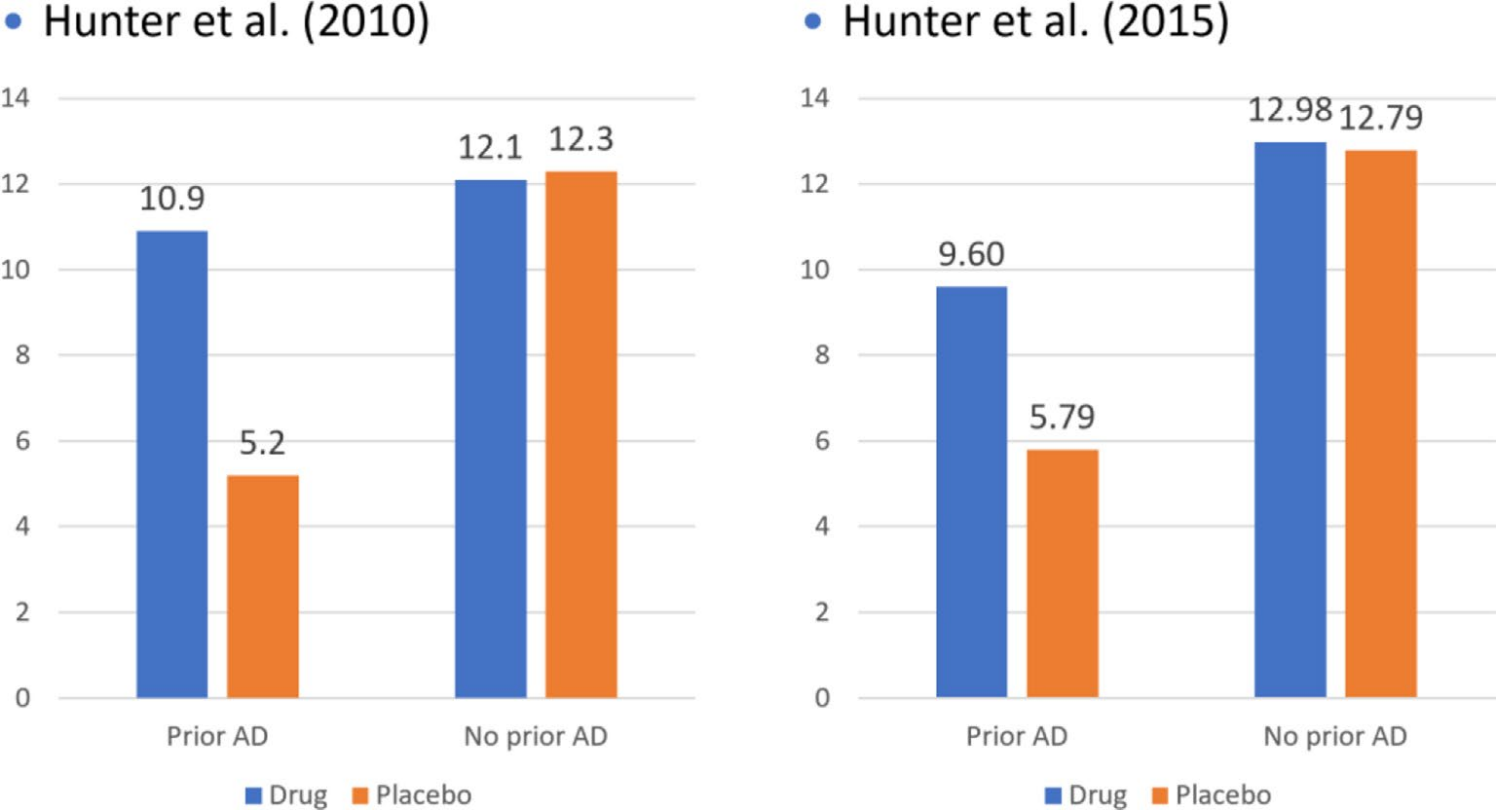
Drug-placebo differences as a function of prior antidepressant use.

Two studies conducted by Aimee Hunter and colleagues at UCLA provide indirect support for this hypothesis (53, 54). In each of these studies, depressed patients in clinical trials were grouped according to whether they had ever been on antidepressants before. As displayed in **Figure 3**, there were virtually no differences at all between drug and placebo among patients who had never been taken antidepressants before. In contrast, among those with prior experience, drug–placebo differences were both significant and substantially larger than those reported in other clinical trials, whereas the combined differences for antidepressant-experienced and antidepressant-naive participants are in the same range of other clinical trials. Taken together, the data from both studies strongly suggest that prescriptions for antidepressants should not be given to depressed people who have never taken them before.

## What Is to Be Done?

How then shall we treat depression? One suggestion that has been made to me informally is to prescribe antidepressants as active placebos. An active placebo is a pharmacologically active substance that does not have specific activity for the condition being treated. Antidepressant medications have little or no pharmacological effects on depression or anxiety, but they do elicit a substantial placebo effect. Could we not use them as a means of capitalizing on the power of placebo?

The problem with this suggestion is that treatment decisions need to be based on an assessment of risks, as well as benefits. The risks of antidepressant treatment include suicidal and violent aggressive behavior in adolescents and young adults; stroke, death from all causes, falls and fractures, and epileptic seizures in the elderly; and sexual dysfunction, withdrawal symptoms, diabetes, deep vein thrombosis, and gastrointestinal and intracranial bleeding in everyone else (55–62). One might argue that these risks might be worth taking for an effective treatment of severe depression, but are they worth risking for a treatment that has no benefit at all over placebo for first-time users?

A second possibility would be to prescribe placebos. They are safe and effective, with relatively few nocebo side effects and no health risks. The problem with prescribing placebos rests with the commonly held assumption that to be effective in clinical practice, placebos have to be presented deceptively as active medications. This assumption has been reported to be false in recent clinical trials [reviewed in Ref. (63)]. In these studies, placebos were presented non-deceptively as placebos with no active ingredients. How could this ever work? The answer is that it was accompanied by a rationale in which it was explained that placebos have been found effective to the condition being treated, that it has been found to involve Pavlovian conditioning, and that it might therefore be effective in treating the person’s condition. This rationale has been found to be critical for the success of the open-label placebo (OLP) intervention (64). Additional OLP trials with larger samples, longer duration, and blinded assessors are warranted.

Unfortunately, only one of the studies assessing OLPs involved the treatment of depression, and that one, although showing promising results, was only a small pilot (65). However, there are many other treatments that equal antidepressants in terms of degree of symptom reduction (66–69). These include psychotherapy, physical exercise, acupuncture, omega-3, homeopathy, tai chi, qigong, and yoga. We do not know the mechanisms of these alternative treatments, and their efficacy may be at least partly due to expectancy, but they are certainly safer than antidepressant medication.

The long-term advantage of psychotherapy over medication has been shown in a number of studies [reviewed in Ref. (70)]. Whereas short-term outcomes were equivalent between the two treatments, long-term outcomes were significantly better for patients who had received psychotherapy than for those who had received medication. Additionally, the National Institute of Mental Health (NIMH) Treatment of Depression Collaborative Research Program reported relapse rates of 36% and 33% for cognitive behaviour therapy and interpersonal therapy, respectively, compared with a 50% relapse rate for antidepressant medication (71). However, the rate of relapse for patients who had recovered on placebo was 33%, the same as that for psychotherapy. There are two take-home messages from these data. First, it dispels the myth that placebo responses are short-lived. Second, it raises the questions of whether psychotherapy reduces relapse or medication increases it (72).

**Figure 4:**
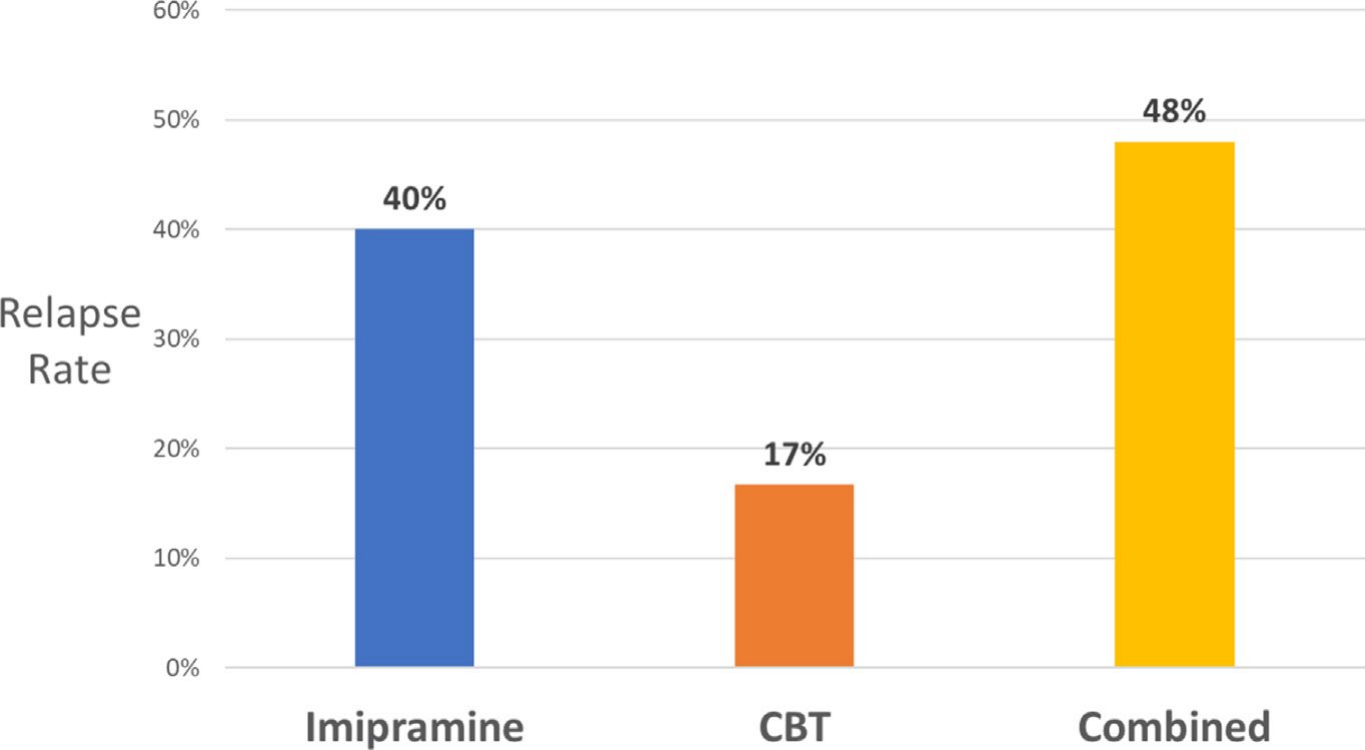
Six-month relapse rates in panic disorder for patients who had been treated with imipramine or placebo, with or without CBT (74).

Support for the hypothesis that antidepressant medication increases the risk of relapse comes from other studies comparing antidepressant and placebo treatment for depression and anxiety disorders. Consistent with the NIMH data, a 2011 meta-analysis reported a relapse rate of 25% for depressed patients successfully treated with placebo compared to relapse rates ranging from 42% to 57% among those treated with various antidepressants (73). A direct test of the effect of antidepressants and psychotherapy on the risk of relapse comes from a study on the treatment of panic disorder (74). The study compared the 6-month relapse rates for patients who had been treated with a tricyclic antidepressant (imipramine), cognitive behavior therapy (CBT), or the two combined. The results, displayed in **Figure 4**, indicate that the risk of relapse following imipramine was more than double that following CBT. However, the addition of the antidepressant to imipramine completely erased that benefit. Similarly, physical exercise as a treatment for depression has been shown to have a much lower relapse rate than SSRIs, but that benefit disappears when the two treatments are combined (75).

These studies reveal another benefit of including placebos in clinical trials of medication. They can reveal situations in which the treatment does more harm than good for the condition being treated. For example, placebos have outperformed antipsychotic medication (haloperidol and risperidone) in the treatment of delirium in palliative care patients and aggression in intellectually disabled adults (76, 77). Similarly, placebo was significantly better than a combination of chondroitin and glucosamine in the treatment of knee osteoarthritis (78) and showed similar superiority in a trial of nutraceuticals in the treatment of depression (79).

Given these data, I suggest that the following principles be used in treatment selection. When treatments are equally effective, recommend the safest. When they are equally safe, let the patient choose which he or she prefers. Before making this choice, however, patients should be accurately informed of the potential harms of antidepressant medication (e.g., increased risk of relapse, suicidality, gastrointestinal and intracranial bleeding, deep vein thrombosis, pulmonary embolism, diabetes, stroke, epilepsy, and death from all causes), as well as the finding that all of these treatments appear to be equally effective in the short term but that psychotherapy and physical exercise might be more effective than antidepressants in the long run.

## Author Contributions

IK wrote the article.

## Conflict of Interest Statement

The author declares that the research was conducted in the absence of any commercial or financial relationships that could be construed as a potential conflict of interest.

The reviewer CB declared a shared affiliation, with no collaboration, with the author to the handling editor.
